# Cytokinin and gibberellic acid-mediated waterlogging tolerance of mungbean (*Vigna radiata* L. Wilczek)

**DOI:** 10.7717/peerj.12862

**Published:** 2022-02-04

**Authors:** M. Rafiqul Islam, Md. Mezanur Rahman, Mohammed Mohi-Ud-Din, Munny Akter, Erin Zaman, Sanjida Sultana Keya, Mehfuz Hasan, Mirza Hasanuzzaman

**Affiliations:** 1 Department of Agronomy, Bangabandhu Sheikh Mujibur Rahman Agricultural University, Gazipur, Bangladesh; 2 Institute of Genomics for Crop Abiotic Stress Tolerance, Department of Plant and Soil Science, Texas Tech University, Lubbock, Texas, United States; 3 Department of Crop Botany, Bangabandhu Sheikh Mujibur Rahman Agricultural University, Gazipur, Bangladesh; 4 Department of Genetic and Plant Breeding, Bangabandhu Sheikh Mujibur Rahman Agricultural University, Gazipur, Bangladesh; 5 Department of Agronomy, Sher-e-Bangla Agricultural University, Dhaka, Bangladesh

**Keywords:** Antioxidants, Hypoxia, Osmoprotectants, Phytohormone, Pulse crop, Photosynthesis, Reactive oxygen species, Yield

## Abstract

**Background:**

Mungbean (*Vigna radiata* L. Wilczek) is one of the most important pulse crops, well-known for its protein-rich seeds. Growth and productivity are severely undermined by waterlogging.

**Methods:**

In this study, we aim to evaluate how two promising phytohormones, namely cytokinin (CK) and gibberellic acid (GA_3_), can improve waterlogging tolerance in mungbean by investigating key morphological, physiological, biochemical, and yield-related attributes.

**Results:**

Our results showed that foliar application of CK and GA_3_ under 5-day of waterlogged conditions improved mungbean growth and biomass, which was associated with increased levels of photosynthetic rate and pigments. Waterlogged-induced accumulation of reactive oxygen species and the consequently elevated levels of malondialdehyde were considerably reduced by CK and GA_3_ treatments. Mungbean plants sprayed with either CK or GA_3_ suffered less oxidative stress due to the enhancement of total phenolics and flavonoids levels. Improvement in the contents of proline and total soluble sugars indicated a better osmotic adjustment following CK and GA_3_ treatments in waterlogged‐exposed plants. Most fundamentally, CK or GA_3_-sprayed waterlogged-stressed mungbean plants demonstrated better performance in the aforementioned parameters after the 15-day recovery period as compared to water-sprayed waterlogged-exposed plants. Our results also revealed that CK and GA_3_ treatments increased yield-associated features in the waterlogged-stressed plant. Here, both phytohormones are efficient in improving mungbean resistance to waterlogging. However, CK was found to be more effective. Overall, our findings suggested that CK or GA_3_ could be used for managing waterlogging-induced damage to mungbean and perhaps in other cash crops.

## Introduction

Mungbean (*Vigna radiata* L. Wilczek) is considered a major source of pulse protein because of its high protein content in the seeds (20.97–31.32%) (USAID Agricultural Extension Support Activity, 2017; (Yi-Shen, Shuai & FitzGerald, 2018; Rahman et al., 2019). More fundamentally, the mungbean is gaining its popularity in the extensive rice-based cropping system, owing to its low fertilizer and pesticide requirements, which provides farmers with good economic benefits and nutritional security (DAE, 2018; Rahman et al., 2019; Hou et al., 2019; Bangladesh Water Development Board, 2020).

Waterlogging has become a major environmental problem in global agriculture (Kaur et al., 2020). Under waterlogged conditions, the mass of oxygen in the soil attenuates partially (hypoxic) or fully (anoxic) due to high microbial activity, and the resulting CO_2_ accumulation in the root zone restricts root metabolism, aerobic respiration, ATP synthesis, and nutrient acquisition, resulting in a significant reduction in the growth, development, and biomass of roots and shoots (Najeeb et al., 2015; Gill et al., 2018; Dossa et al., 2019). Moreover, waterlogging impairs root permeability and causes root injury, leading to a decrement of hydraulic conductivity and consequent stomatal closure, resulting in a significant reduction in net photosynthetic and transpiration rates (Else et al., 2001; Anee et al., 2019). These also lead to the excess generation of reactive oxygen species (ROS) such as superoxide (O_2_^•−^), singlet oxygen (^1^O_2_), hydrogen peroxide (H_2_O_2_) and hydroxyl radical (^•^OH), which are harmful to biological macromolecules (Yeung et al., 2018; Zhou et al., 2020).

Plants can acclimatize to the adverse impact of waterlogging-mediated soil oxygen dearth through adapting various morphological, physiological, and biochemical mechanisms. The development of adventitious roots and faster stem elongation are some examples of morphological adaptations (Yamauchi et al., 2018), whereas reduced stomatal conductance and subsequent decrease in net photosynthetic rate are short-term physiological adaptations (Jacobsen et al., 2007; Bhusal et al., 2020). Additionally, to shield themselves from stress, waterlogged plants accumulate several osmoprotectants such as proline, soluble sugars, and sucrose (Tewari & Mishra, 2018). However, one of the most important adaptive mechanisms is a well-balanced antioxidant defense system that involves both enzymatic (*e.g*., superoxide dismutase, SOD; catalase, CAT; ascorbate peroxidase, APX; glutathione *S*-transferase, GST; glutathione peroxidase, GPX) and non-enzymatic (*e.g*., total phenolics, total flavonoids, carotenoids) antioxidants that scavenge overaccumulation of ROS (Garcia et al., 2020; Hasanuzzaman et al., 2020).

Since mungbean is a waterlogging sensitive crop, there must devise a strategy for effectively alleviating the destructive effect of waterlogging stress to improve mungbean production in flood-prone areas having intensive and erratic rains. In this context, the search for effective approaches propelled us to use phytohormones such as cytokinin (CK) and gibberellic acid (GA_3_) for overcoming the problems associated with waterlogging.

Although there are several studies on the role of phytohormones on abiotic stress tolerance in crop plants, there are hardly any studies focusing on the coordinated actions of CK and GA_3_ in modulating plant growth and physiology. In the present study, we examined whether CK and GA_3_ protect mungbean from waterlogging damage. We also examined the regulatory roles of CK and GA_3_ in improving waterlogging tolerance of mungbean by investigating the morpho-physiological and biochemical mechanisms through assessments of morpho-physiology, photosynthetic parameters, ROS metabolism, osmoregulation, and yield.

## Materials and Methods

### Plant growth conditions

Mungbean genotype, VC6173-B, was used in the present study owing to their properties of short duration (80 days to maturity), greater grain weight (44 g/1,000 grain), and high yield potential (1.5 t ha^−1^). Healthy seeds of VC6173-B were germinated following the procedures of Rahman et al. (2019). Afterward, six germinated seeds were sown in each plastic pot (height × diameter = 20 cm × 16 cm), having 8 kg of silt loam soil. The soil was fertilized as per the recommended dose (Ahmmed et al., 2018). Plants were kept free from pests and diseases. The average maximum and minimum temperature of the experimental area ranged from 32.6 to 22.8 °C, with 85.6% of relative humidity. The number of seedlings was thinned to two in each pot after the eighth day of germination and continues to grow in normal condition up to the imposition of treatments. The experiment was laid out in a completely randomized design (CRD) with four replications.

### Imposition of waterlogging treatments

Before waterlogging exposure for the next 5 days, 15-day-old mungbean seedlings at the V_1_ stage (when the first trifoliate were fully developed) were grouped into four sets as (i) water-sprayed waterlogged-stress-free plants (Control), (ii) water-sprayed waterlogged-stressed plants (WL), (iii) cytokinin (CK), named kinetin-sprayed (50 mg L^−1^) waterlogged-stressed plants (CK+WL) and (iv) gibberellic acid (GA_3_)-sprayed (50 mg L^−1^) waterlogged-stressed plants (GA_3_+WL). It is worth noting that foliar spray with either CK or GA_3_ or water (20 mL to each pot) was done two times daily (9.00 to 10.00 am, and 3.00 to 4.00 pm). Tween-20 surfactant (0.2%, v/v) was used to ensure maximum adherence of CK, GA_3_, and water to the leaves. Notably, the level of water for creating waterlogging stress was maintained at 2.5 cm above the soil surface. Following the stress period, one set of seedlings (Set I) was immediately harvested, and another set (Set II) was harvested at 15-day after recovery to record the morpho-physiological and biochemical parameters. After stress exposure, one set of seedlings (Set III) was allowed to grow until harvest with optimum irrigation to determine yield-related attributes. Importantly, for determining various morphological, physiological, and biochemical parameters, the first trifoliate leaves from the bottom of the plants were collected.

### Determination of growth-related parameters

The growth performance of mungbean plants was assessed by measuring the shoot height, and dry weight (DW) of both shoots and roots. Additionally, the stem girth of the mungbean plants was determined using a slide caliper.

### Measurement of photosynthetic parameters

Gas exchange features, including photosynthesis rate (*Pn*), stomatal conductance to water (H_2_O) (*g*_*s*_), and transpiration rate (*E*) of mungbean plants were measured using the LI-6400XT portable photosynthesis system (LI-COR Biosciences, Lincoln, NE, USA) from 11.00 AM to 2.00 PM under full sun-light conditions. The parameters *Pn*, *g*_*s*,_ and *E* were used for assessing the instantaneous water-use efficiency (WUEins; ratio *Pn*/*E*) and intrinsic water-use efficiency (WUEint; ratio *Pn*/*g*_*s*_). The freshly harvested mungbean leaves were used to determine the contents of chlorophylls (Chls) [Chl *a*, Chl *b* and Chl (*a*+*b*)] by using the visible spectrophotometer (Model: T60 UV, PG Instruments Limited, Leicestershire, UK) according to the protocols described by Lichtenthaler & Wellburn (1983). The contents of total phenolic and total flavonoid were quantified following the methods of Ainsworth & Gillespie (2007), and Zhishen, Mengcheng & Jianming (1999), respectively.

### Measurement of oxidative stress indicators

Fresh leaf tissues (0.5 g) were homogenized in 3 mL of 5% (w/v) trichloroacetic acid (TCA). After centrifugation at 11,500× *g* for 10 min, the supernatant was used to determine H_2_O_2_ and malondialdehyde (MDA). The H_2_O_2_ content was determined spectrophotometrically according to the procedure of Yu, Murphy & Lin (2003) with some modifications. Briefly, 400 µL supernatant was added to 400 mL of 10 mM potassium phosphate buffer (pH 7.0) and 800 mL of 1 M potassium iodide (KI). The reaction was allowed to proceed in the dark for 1 h before measuring the absorbance at 390 nm. The H_2_O_2_ concentration was calculated using the extinction coefficient of 0.28 μM^−1^ cm^−1^. The methods of Heath & Packer (1968) were followed for MDA determination. The MDA content was calculated using an extinction coefficient of 155 mM^−1^ cm^−1^ and represented as nmol g^−1^ fresh weight (FW).

### Quantification of proline and soluble sugar content

Leaf proline content was measured spectrophotometrically by an acid-ninhydrin method using the procedure outlined by Bates, Waldren & Teare (1973). The proline content was calculated using a standard curve and reported as µmol g^−1^ FW. Freshly harvested leaf tissues were used for the quantification of total soluble sugar levels following the comprehensive protocols described by Somogyi (1952).

### Statistical analysis

The obtained data were subjected to a one-way analysis of variance (ANOVA) using Statistix software (version 10). Different alphabetical letters were used to denote the significant variations among different treatments at the *P* < 0.05 level following a least significant difference (LSD) test. All the numerical data in figures and tables are presented as means ± standard errors (SEs) of four independent replications.

## Results

### CK and GA_3_ supplementation improved growth parameters of mungbean plants during waterlogging stress and recovery period

In comparison with control plants, WL plants displayed a significant decrease in shoot height, shoot DW, root DW, and stem girth by 34%, 37%, 42%, and 37%, respectively; however, following 15-day of recovery, these parameters were reduced by 35%, 44%, 36% and 32%, respectively (Table 1). On the other hand, CK+WL and GA_3_+WL plants showed noteworthy improvement in shoot height by 36% and 26%, shoot DW by 38% and 27%, root DW by 36% and 26%, and stem girth by 36% and 31%, respectively, when compared with WL plants (Table 1). Following the recovery period, a substantial enhancement in shoot height (35% and 24%), shoot DW (38% and 27%), root DW (36% and 26%), and stem girth (36% and 31%) was observed in the CK+WL and GA_3_+WL plants, respectively, in comparison with WL plants (Table 1).

**Table 1:** The effects of exogenous cytokinin and gibberellic acid on shoot height, shoot dry weight, root dry weight and stem girth width of mungbean plants after 5-day of stress and 15-day of recovery periods.

Treatments	Shoot height (cm)		Shoot DW (g)		Root DW (g)		Stem girth width (mm)	
	After stress	After recovery	After stress	After recovery	After stress	After recovery	After stress	After recovery
Control	16.96 ± 0.65^a^	33.21 ± 1.58^a^	2.34 ± 0.09^a^	9.68 ± 0.44^a^	0.37 ± 0.02^a^	1.90 ± 0.07^a^	4.82 ± 0.31^a^	7.04 ± 0.04^a^
WL	11.29 ± 0.42^c^	21.49 ± 0.90^d^	1.47 ± 0.09^c^	5.42 ± 0.17^c^	0.22 ± 0.01^c^	1.22 ± 0.10^c^	3.03 ± 0.14^c^	4.77 ± 0.27^c^
CK+WL	15.29 ± 0.46^b^	28.94 ± 0.58^b^	2.03 ± 0.06^b^	7.34 ± 0.26^b^	0.29 ± 0.01^b^	1.65 ± 0.06^ab^	4.12 ± 0.08^b^	6.41 ± 0.20^b^
GA_3_+WL	14.17 ± 0.33^b^	26.62 ± 0.48^c^	1.87 ± 0.03^b^	6.78 ± 0.30^b^	0.27 ± 0.01^b^	1.51 ± 0.12^bc^	3.96 ± 0.12^b^	5.92 ± 0.18^b^

**Note:**

Values are means ± standard errors (*n* = 4). Different alphabetical letters as superscripted within the same column indicate significant differences among various treatments according to a least significant difference test (LSD) at *P* < 0.05. WL, water-sprayed waterlogged-stressed plants; CK+WL, cytokinin (CK)-sprayed (50 mg L^−1^) waterlogged-stressed plants; GA_3_+WL, gibberellic acid (GA)-sprayed (50 mg L^−1^) waterlogged-stressed plants; DW, dry weight.

### Exogenous CK and GA_3_ enhanced gas exchange features of mungbean plants during waterlogging stress and recovery period

**Figure 1:**
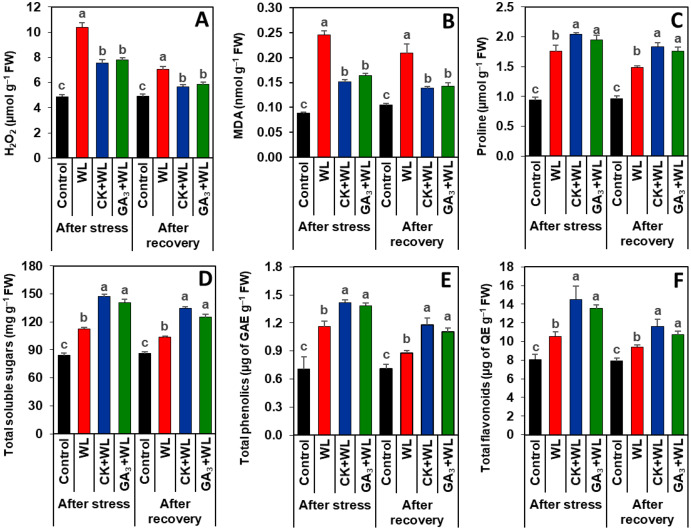
Effects of exogenous cytokinin and gibberellic acid on (A) photosynthetic rate, (B) conductance to water, (C) transpiration rate (D) WUEint and (E) WUEins in the leaves of mungbean plants after 5-day of stress and 15-day of recovery period. Data represent means of four independent replicates (*n* = 4). Vertical bars indicate standard errors. Different letters represent significant differences at *P* < 0.05 (least significant difference test). WL, water-sprayed waterlogged-stressed plants; CK+WL, cytokinin (CK)-sprayed (50 mg L^−1^) waterlogged-stressed plants; GA_3_+WL, gibberellic acid (GA)-sprayed (50 mg L^−1^) waterlogged-stressed plants; WUEint, intrinsic water-use efﬁciency; WUEins, instantaneous water-use efﬁciency.

Plants subjected to waterlogging stress and subsequent recovery period showed a decrease in *Pn* (by 44% and 42%), *gs* (90% and 53%) and *E* (74% and 36%, respectively) when compared with control plants (Figs. 1A–1C). Nonetheless, the levels of WUEint and WUEins in WL plants increased by 515% and 118%, respectively relative to control plants, whereas the level of WUEint and WUEins in control and WL plants was comparable after the recovery period (Figs. 1D, 1E). On the other hand, CK+WL and GA_3_+WL plants exhibited decreased levels of *gs* (by 40% and 27%) and *E* (33% and 23%), and increased levels of *Pn* (by 37% and 27%), WUEint (119% and 64%) and WUEins (100% and 61%, respectively) when compared with that of WL plants (Figs. 1A–1E). Similarly, following the recovery period, decreased levels of *gs* (by 36% and 28%) and *E* (34% and 21%), and increased levels of *Pn* (37% and 27%), WUEint (115% and 73%), and WUEins (107% and 59%) were recorded in CK+WL and GA_3_+WL plants, respectively, as compared with that of WL plants (Figs. 1A–1E).

### CK and GA_3_ prevented damage to photosynthetic pigments during waterlogging stress and recovery period

**Figure 2:**
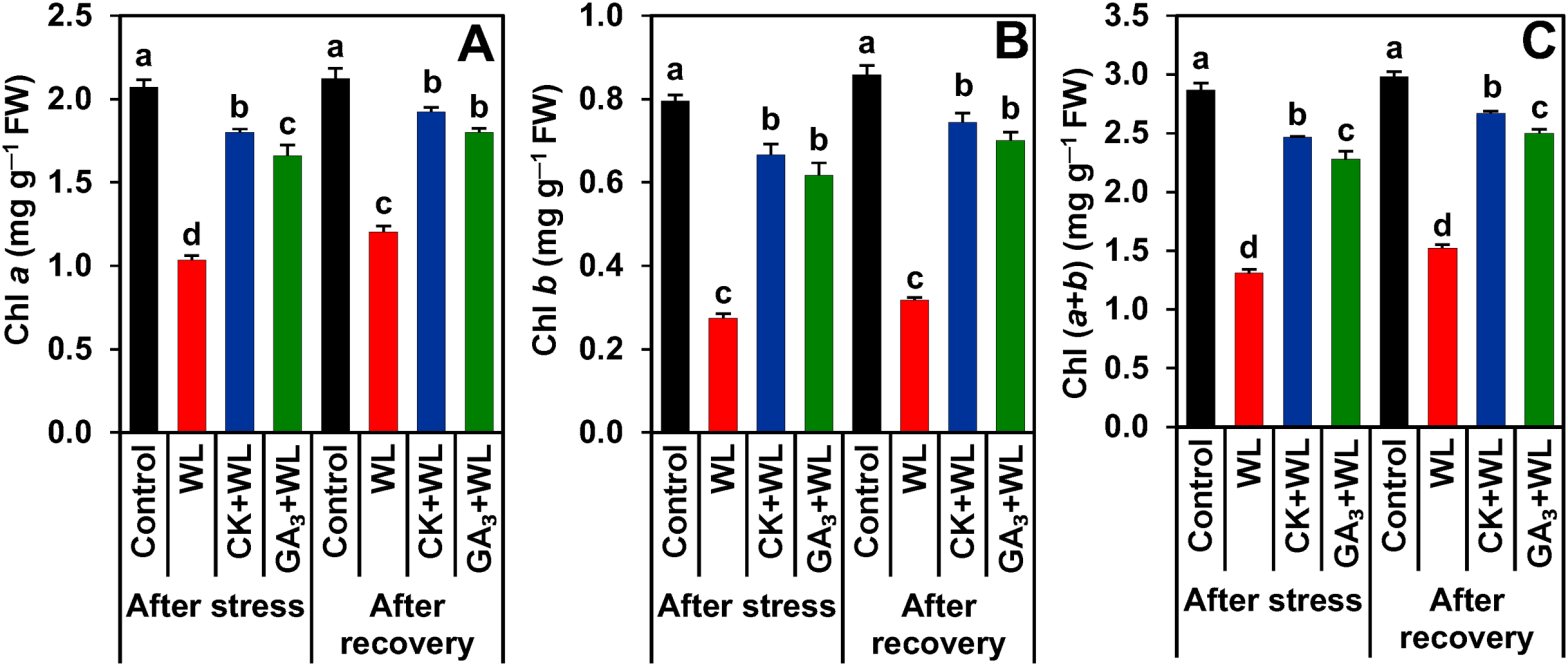
Effects of exogenous cytokinin and gibberellic acid on (A) Chlorophyll (Chl) *a*, (B) Chl *b* and (C) Chl (*a* + *b*) in the leaves of mungbean plants after 5-day of stress and 15-day of recovery period. Data represent means of four independent replicates (*n* = 4). Vertical bars indicate standard errors. Different letters represent significant differences at *P* < 0.05 (least significant difference test). WL, water-sprayed waterlogged-stressed plants; CK+WL, cytokinin (CK)-sprayed (50 mg L^−1^) waterlogged-stressed plants; GA_3_+WL, gibberellic acid (GA)-sprayed (50 mg L^−1^) waterlogged-stressed plants; FW, fresh weight.

Compared to control plants, a sharp decline in the contents of Chl *a* (by 50.10% and 43%), Chl *b* (65.44% and 63%), and Chl (*a*+*b*) (54% and 49%) were observed in the leaves of WL plants following stress and recovery periods, respectively (Figs. 2A–2C). In contrast, CK and GA_3_ supplementation protected the photosynthetic pigments from waterlogged-induced deleterious effects by enhancing the contents of Chl *a* (by 74% and 61%), Chl *b* (142% and 124%), and Chl (*a*+*b*) (88% and 74%) in the leaves of CK+WL and GA_3_+WL plants, respectively (Figs. 2A–2C). Furthermore, following the recovery period, the leaves of CK+WL and GA_3_+WL plants also displayed a significant rise in the content of Chl *a* (by 60% and 50%), Chl *b* (134% and 120%), and Chl (*a*+*b*) (75% and 64%, respectively) in comparison with WL plants (Figs. 2A–2C).

### Exogenous CK and GA_3_ suppressed oxidative stress markers in mungbean plants during waterlogging stress and recovery period

**Figure 3:**
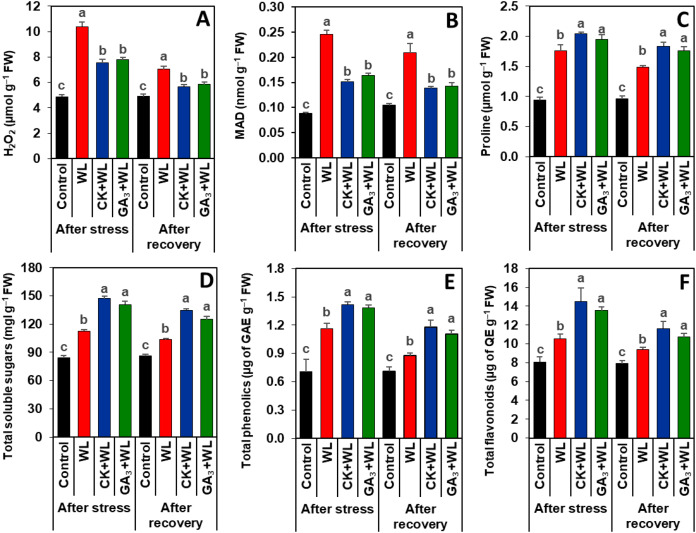
Effects of exogenous cytokinin and gibberellic acid on the contents of (A) H_2_O_2_, (B) MDA, (C) proline, (D) total soluble sugars, (E) total phenolics and (F) total flavonoids in the leaves of mungbean plants after 5-day of stress and 15-day of recovery period. Data represent means of four independent replicates (*n* = 4). Vertical bars indicate standard errors. Different letters represent significant differences at *P* < 0.05 (least significant difference test). WL, water-sprayed waterlogged-stressed plants; CK+WL, cytokinin (CK)-sprayed (50 mg L^−1^) waterlogged-stressed plants; GA_3_+WL, gibberellic acid (GA)-sprayed (50 mg L^−1^) waterlogged-stressed plants; FW, fresh weight; GAE, gallic acid equivalent; H_2_O_2_, hydrogen peroxide; QE, quercetin equivalent, malondialdehyde.

Imposition of waterlogging stress and following recovery period substantially increased the contents of H_2_O_2_ (by 113% and 62%) and MDA (179% and 100%, respectively) in the leaves of WL plants, relative to that of control plants (Figs. 3A, 3B). CK and GA_3_ treatment resulted in reductions of H_2_O_2_ (by 57% and 45%) and MDA (29% and 19%) contents in the leaves of CK+WL and GA_3_+WL plants, respectively, in comparison with WL plants (Figs. 3A, 3B). Similarly, compared with the WL plants, substantial decreases in the contents of H_2_O_2_ (by 37% and 26%) and MDA (34% and 21%) were noticed in the leaves of CK+WL and GA_3_+WL plants, respectively, in the recovery period (Figs. 3A, 3B).

### CK and GA_3_ enhanced the synthesis of osmolytes and non-enzymatic antioxidants in mungbean plants during waterlogging stress and recovery period

Exposure of mungbean plants to waterlogging stress and followed by a recovery period exhibited a rise in the contents of proline (63% and 42%), total soluble sugars (33% and 20%), total phenolics (58% and 23%), and total flavonoids (31% and 16%, respectively) in the leaves of WL plants when compared with that of control plants (Figs. 3C–3F). In comparison with WL plants, CK+WL and GA_3_+WL plants displayed enhanced levels of proline (by 34% and 21%), total soluble sugars (32% and 22%), total phenolics (36% and 24%), and total flavonoids (37% and 28%, respectively) (Figs. 3C–3F). Similarly, in the WL plants, the levels of proline, total soluble sugars, total phenolics, and flavonoids were noticeably enhanced in CK+WL leaves by 34%, 35%, 34%, and 35%, respectively, and in GA_3_+WL leaves by 23%, 27%, 23% and 25%, respectively, followed by a recovery period (Figs. 3C–3F).

### CK and GA_3_ supplementation increased seed yield of mungbean plants

Compared to the control, WL plants displayed notable decrements in pod length (by 13%), the total number of pods per plant (36%), thousand seed weight (13%) and seed yield per plant (42%) (Table 2). Significant improvement in pod length (by 8% and 7%), the total number of pods per plant (19% and 16%), and seed yield per plant (20% and 17%) were observed in CK+WL and GA_3_+WL plants, respectively, when compared with that of WL plants (Table 2).

**Table 2:** The effects of exogenous cytokinin and gibberellic acid on yield-contributing parameters of stressed and stress-free mungbean plants.

Treatments	Pod length (cm)	Total number of pods per plant	Thousand seed weight (g)	Seed yield (g per plant)
Control	8.46 ± 0.19^a^	33.00 ± 1.29^a^	51.10 ± 0.86^a^	16.47 ± 0.59^a^
WL	7.35 ± 0.19^c^	21.00 ± 0.41^c^	44.21 ± 1.07^b^	9.51 ± 0.30^c^
CK+WL	8.03 ± 0.10^ab^	26.00 ± 1.47^b^	47.12 ± 2.31^ab^	11.94 ± 0.64^b^
GA_3_+WL	7.92 ± 0.09^b^	25.00 ± 0.41^b^	46.23 ± 1.88^ab^	11.49 ± 0.33^b^

**Note:**

Values are means ± standard errors (*n* = 4). Different alphabetical letters as superscripted within the same column indicate significant differences among various treatments according to a least significant difference test (LSD) (*P* < 0.05). WL, water-sprayed waterlogged-stressed plants; CK+WL, cytokinin (CK)-sprayed (50 mg L^−1^) waterlogged-stressed plants; GA_3_+WL, gibberellic acid (GA)-sprayed (50 mg L^−1^) waterlogged-stressed plants.

## Discussion

Waterlogging leads to detrimental consequences on a number of crop plants, including mungbean (Amin et al., 2015; Donat et al., 2016). The current study investigated the effective mitigation of waterlogged-induced damage to mungbean plants by exploring the potential role of CK and GA_3_.

In the present study, waterlogging-induced deleterious effects were evident as a reduction of shoot height, shoot and root DW, and stem girth; however, the foliar application of CK and GA_3_ partially alleviated those waterlogged-mediated detrimental effects. It is well reported that both CK and GA_3_ played a pivotal role in cell elongation and other growth and development processes (Ahanger et al., 2018; Wang et al., 2019; Rady et al., 2021), which resulted in enhanced growth and biomass of mungbean under waterlogging in the present study.

The waterlogged-induced growth inhibition and biomass reduction in mungbean plants might be a consequence of impeded photosynthesis. It is envisaged that at the beginning of waterlogging stress, plant roots rapidly transmit a xylem-borne signal to the leaves in the form of hormones, most notably abscisic acid (ABA) to slow down the process of transpiration through stomatal closure, and thus attenuate carbon dioxide availability in leaves (Jackson et al., 2003; Najeeb et al., 2015). This was due to the decline in root hydraulic conductivity and inadequate ATP production during waterlogging as reported by other researchers (Steffens et al., 2005; Kaur et al., 2019). Henceforth, the supply of photoassimilates from leaves to different plant parts is also reduced, which ultimately leads to poor plant growth and biomass production (Kogawara et al., 2006). Apart from these, waterlogged-induced reduction of photosynthetic pigments, including Chl *a* and *b*, is also responsible for reduced net photosynthetic rate (Ren et al., 2016; Zhang et al., 2020), as also observed in the present study (Figs. 1A and 2A–2C). On the other hand, mungbean plants treated with CK and GA_3_ significantly improved the net photosynthetic, as well as the contents of Chl *a* and Chl *b* under waterlogged conditions, implying that CK and GA_3_ played a pivotal role in improving the photosynthetic process during waterlogging stress. Improvements in photosynthesis, as well as photosynthetic pigments, were also observed after the recovery period in response to CK and GA_3_ supplementation. This was due to the fact that phytohormones could regulate photoprotection of the photosynthetic apparatus under stress conditions and they controlled the production and scavenging of photosynthesis-derived ROS, the duration and extent of photo-oxidative stress, and redox signaling (Müller & Munné-Bosch, 2021).

We also observed that in comparison with WL plants, CK or GA_3_-treated plants demonstrated remarkably improved WUE, indicating that CK and GA_3_ might enable mungbean plants to produce more biomass under the conditions of limited water uptake. Surprisingly, after the recovery period, we have seen similar positive effects of CK and GA_3_ in boosting WUE. It is well-known that improving WUE in abiotic-stressed plants without deteriorating growth and yield is scrutinized as a paramount goal of current plant breeding programs (Yang et al., 2016). The increase in WUE was mainly due to the enhancement of photosynthetic parameters. Our findings support this phenomenon and corroborate with the previous findings where the external application of CK to cold-stressed coffee (*Coffea arabica*) and GA_3_ to drought-stressed *Vicia faba* seedlings improved WUE (Acidri et al., 2020; Rady et al., 2021).

Water stagnation because of waterlogging conditions causes poor gas exchange between soils and plant roots, resulting in hypoxia or anoxia in plant tissues (Matin & Jalali, 2017). The scarcity of oxygen induces leakage of electrons from mitochondria, which together with impaired photosynthesis, triggers a burst of excessive ROS production, causing oxidative damage to waterlogged-stressed seedlings (Xu et al., 2013), and was manifested in our study with increasing H_2_O_2_ and MDA in WL plants (Figs. 3A, 3B). External application of either CK or GA_3_ to waterlogged-stressed mungbean plants resulted in a dramatic reduction in ROS accumulation, implying that CK and GA_3_ play an important role in reducing oxidative burden induced by ROS and providing protection to plasma membrane integrity. It is well established that phytohormones controlled the production and scavenging of photosynthesis-derived ROS and also enhanced antioxidant defense (Müller & Munné-Bosch, 2021). In line with our study, previous reports also demonstrated that the contents of H_2_O_2_ and MDA were noticeably diminished in salt-stressed tomato (*Solanum lycopersicum*) and okra (*Abelmoschus esculentus*) by CK application, and drought-stressed wheat (*Triticum aestivum*) and boron-stressed tomato by GA_3_ application (Ahanger et al., 2018; Wang et al., 2019; Moumita et al., 2019; Javed et al., 2021).

Our results also demonstrated that, compared with WL, the leaves of CK+WL and GA_3_+WL plants accumulated higher levels of non-enzymatic antioxidants, namely total phenolics and total flavonoids (Figs. 3E, 3F). More fundamentally, following the recovery period, there was a similar pattern of improvement in total phenolics and total flavonoids levels in response to CK or GA_3_ application. Phenolics and flavonoids are two well-known secondary metabolites that play a pivotal role in scavenging free radicals and preventing lipid peroxidation, thus maintaining membrane fluidity and shielding cell membrane damage from oxidative damage under different abiotic stresses, including waterlogging (Ahmad et al., 2010; Patel et al., 2020; Elkelish et al., 2020). These results implied that either CK or GA_3_-induced alleviation of ROS burden was most likely due to the heightened level of non-enzymatic antioxidants. It is worth noting that the application of CK to mungbean plants displayed a greater role in alleviating the contents of H_2_O_2_ and MDA by enhancing the levels of total phenolics and flavonoids when compared with the plants treated with GA_3_. Ahanger et al. (2018) reported that CK application to salt-stressed tomato plants increased the level of total phenolics and flavonoids, while Rady et al. (2021) revealed that GA_3_ supplementation to drought-stressed faba bean plants increased phenolics accumulation.

To overcome waterlogged-induced osmotic stress, plants also accumulate several compatible solutes, including proline and total soluble sugars, which play vital roles in maintaining water balance, retrieving photosynthetic functions, stabilizing cellular components, ROS scavenging, improving cellular signaling, and secondary metabolite biosynthesis (Barickman, Simpson & Sams, 2019; Chávez-Arias, Gómez-Caro & Restrepo-Díaz, 2019; Xiao et al., 2020). Our data on compatible solutes revealed that the levels of proline and total soluble sugar were dramatically enhanced in WL plants (Figs. 3C, 3D). Importantly, exogenous application of CK and GA_3_ further enhanced the contents of proline and total soluble sugars in the leaves of CK+WL and GA_3_+WL plants, and this trend was similar following the recovery period (Figs. 3C, 3D). Our findings coincided with the results of Sarafraz-Ardakani et al. (2014), who also observed that CK supplementation increased the amount of proline and soluble sugars in drought-stressed wheat plants.

Waterlogged-stressed mungbean plants showed a decrease in yield-associated features such as pod length, the total number of pods per plant, thousand seed weight, and seed yield per plant, which might be correlated with poor plant growth because of impaired photosynthesis and enhanced accumulation of ROS (Kumar et al., 2013). In contrast, foliar application of either CK or GA_3_ to waterlogged-exposed mungbean plants resulted in a dramatic increase in pod length, the total number of pods per plant, thousand seed weight, and seed yield per plant. These results suggested that CK or GA_3_ supplementation improved photosynthetic efficiency of mungbean to sustain higher levels of total soluble sugars, as well as heightened levels of proline and non-enzymatic antioxidants to resist the phytotoxic effects of waterlogging through the reductions of ROS-mediated oxidative stress, resulting in improvement of yield performance. Yield improvement in wheat plants under heat and drought stress was also reported upon supplementation of CK and GA_3_, respectively (Gupta, Agarwal & Gupta, 2012*; Yang et al., 2016*).

## Conclusions

Our work revealed that treating plants with either CK or GA_3_ positively regulates defense responses in waterlogged-stressed mungbean plants through regulating several physiological and biochemical mechanisms. The CK and GA_3_-sprayed plants demonstrated improvement in plant height, stem girth, and plant biomass and a decreased level of ROS accumulation and consequent MDA level, implying that CK and GA_3_ played a decisive role in controlling ROS-induced cellular damage. These observations were closely interlinked to heightened levels of non-enzymatic antioxidants, including total phenolics and total flavonoids. Elevated levels of proline and total soluble sugars in CK and GA_3_-sprayed plants also contributed to the maintenance of better water status and osmotic adjustment under waterlogging. Such positive regulatory roles of CK and GA_3_ also persisted after the 15-day recovery period. All the studied physiological and biochemical mechanisms in CK and GA_3_-sprayed plants contribute to improving the yield-associated features of mungbean plants.

These findings imply that CK and GA_3_ could be used as a potential chemicals for reducing the negative impacts of waterlogging on crops and on agricultural production to ensuring the sustainability of agriculture in flood-prone areas.

## Supplemental Information

10.7717/peerj.12862/supp-1 Supplemental Information 1 Raw data.

## Additional Information and Declarations

### Data Availability

The following information was supplied regarding data availability: The raw data are available in the Supplemental File.

## References

[ref-1] Acidri R, Sawai Y, Sugimoto Y, Handa T, Sasagawa D, Masunaga T, Yamamoto S, Nishihara E (2020). Exogenous kinetin promotes the nonenzymatic antioxidant system and photosynthetic activity of coffee (*Coffea arabica* L.) plants under cold stress conditions. Plants.

[ref-2] Ahanger MA, Alyemeni MN, Wijaya L, Alamri SA, Alam P, Ashraf M, Ahmad P (2018). Potential of exogenously sourced kinetin in protecting *Solanum lycopersicum* from NaCl-induced oxidative stress through up-regulation of the antioxidant system, ascorbate-glutathione cycle and glyoxalase system. PLOS ONE.

[ref-3] Ahmad P, Jaleel CA, Salem MA, Nabi G, Sharma S (2010). Roles of enzymatic and non-enzymatic antioxidants in plants during abiotic stress. Critical Reviews in Biotechnology.

[ref-4] Ahmmed S, Jahiruddin M, Razia S, Begum RA, Biswas JC, Rahman ASMM (2018). Fertilizer recommendation guide-2018.

[ref-5] Ainsworth EA, Gillespie KM (2007). Estimation of total phenolic content and other oxidation substrates in plant tissues using Folin-Ciocalteu reagent. Nature Protocols.

[ref-6] Amin MR, Karim MA, Khaliq QA, Islam MR, Aktar S (2015). Effect of nitrogen and potassium on the root growth, nutrient content and yield of mungbean (*Vigna radiata*) under waterlogged condition. Agriculturists.

[ref-7] Anee TI, Nahar K, Rahman A, Mahmud JA, Bhuiyan TF, Alam MU, Fujita M, Hasanuzzaman M (2019). Oxidative damage and antioxidant defense in (*Sesamum indicum*) after different waterlogging durations. Plants.

[ref-8] Bangladesh Water Development Board (2020). Flood inundation map of Bangladesh, flood forecasting & warning centre, Bangladesh. http://www.ffwc.gov.bd/index.php/map/inundation-map/bangladesh-today.

[ref-9] Barickman TC, Simpson CR, Sams CE (2019). Waterlogging causes early modification in the physiological performance, carotenoids, chlorophylls, proline, and soluble sugars of cucumber plants. Plants.

[ref-10] Bates LS, Waldren RP, Teare ID (1973). Rapid determination of free proline for water-stress studies. Plant and Soil.

[ref-11] Bhusal N, Kim HS, Han SG, Yoon TM (2020). Photosynthetic traits and plant-water relations of two apple cultivars grown as bi-leader trees under long-term waterlogging conditions. Environmental and Experimental Botany.

[ref-12] Chávez-Arias CC, Gómez-Caro S, Restrepo-Díaz H (2019). Physiological, biochemical and chlorophyll fluorescence parameters of *Physalis peruviana* seedlings exposed to different short-term waterlogging periods and Fusarium wilt infection. Agronomy.

[ref-13] DAE (2018). Krishi dairy. Agricultural information service.

[ref-14] Donat MG, Lowry AL, Alexander LV, O’Gorman PA, Maher N (2016). More extreme precipitation in the world’s dry and wet regions. Nature Climate Change.

[ref-15] Dossa K, You J, Wang L, Zhang Y, Li D, Zhou R, Yu J, Wei X, Zhu X, Jiang S, Gao Y, Mmadi MA, Zhang X (2019). Transcriptomic profiling of sesame during waterlogging and recovery. Scientific Data.

[ref-16] Elkelish AA, Alhaithloul HAS, Qari SH, Soliman MH, Hasanuzzaman M (2020). Pretreatment with *Trichoderma harzianum* alleviates waterlogging-induced growth alterations in tomato seedlings by modulating physiological, biochemical, and molecular mechanisms. Environmental and Experimental Botany.

[ref-17] Else MA, Coupland D, Dutton L, Jackson MB (2001). Decreased root hydraulic conductivity reduces leaf water potential, initiates stomatal closure and slows leaf expansion in flooded plants of castor oil (*Riccinus communis*) despite diminished delivery of ABA from the roots to shoots in the xylem sap. Physiologia Plantarum.

[ref-18] Garcia N, da-Silva CJ, Cocco KLT, Pomagualli D, de Oliveira FK, da Silva JVL, de Oliveria ACB, do Amarante L (2020). Waterlogging tolerance of five soybean genotypes through different physiological and biochemical mechanisms. Environmental and Experimental Botany.

[ref-19] Gill MB, Zeng F, Shabala L, Böhm J, Zhang G, Zhou M, Shabala S (2018). The ability to regulate voltage-gated K^+^-permeable channels in the mature root epidermis is essential for waterlogging tolerance in barley. Journal of Experimental Botany.

[ref-20] Gupta S, Agarwal VP, Gupta NK (2012). Efficacy of putrescine and benzyladenine on photosynthesis and productivity in relation to drought tolerance in wheat (*Triticum aestivum*). Physiology and Molecular Biology of Plants.

[ref-21] Hasanuzzaman M, Bhuyan MHMB, Zulfiqar F, Raza A, Mohsin SM, Mahmud JA, Fujita M, Fotopoulos V (2020). Reactive oxygen species and antioxidant defense in plants under abiotic stress: revisiting the crucial role of a universal defense regulator. Antioxidants.

[ref-22] Heath RL, Packer L (1968). Photoperoxidation in isolated chloroplasts: I. Kinetics and stoichiometry of fatty acid peroxidation. Achives of Biochemistry and Biophysics.

[ref-23] Hou D, Yousaf L, Xue Y, Hu J, Wu J, Hu X, Feng N, Shen Q (2019). Mungbean (*Vigna radiata*): bioactive polyphenols, polysaccharides, peptides, and health benefits. Nutrients.

[ref-24] Jackson MB, Saker LR, Crisp CM, Else MA, Janowiak F (2003). Ionic and pH signalling from roots to shoots of flooded tomato plants in relation to stomatal closure. Plant and Soil.

[ref-25] Jacobsen AL, Agenbag L, Esler KJ, Pratt RB, Ewers FW, Davis SD (2007). Xylem density, biomechanics and anatomical traits correlate with water stress in 17 evergreen shrub species of the Mediterranean-type climate region of South Africa. Journal of Ecology.

[ref-26] Javed T, Ali MM, Shabbir R, Anwar R, Afzal I, Mauro RP (2021). Alleviation of copper-induced stress in pea (*Pisum sativum*) through foliar application of gibberellic acid. Biology.

[ref-27] Kaur G, Singh G, Motavalli PP, Nelson KA, Orlowski JM, Golden BR (2020). Impacts and management strategies for crop production in waterlogged or flooded soils: a review. Agronomy Journal.

[ref-28] Kaur G, Zurweller B, Motavalli PP, Nelson KA (2019). Screening corn hybrids for soil waterlogging tolerance at an early growth stage. Agriculture.

[ref-29] Kogawara S, Yamanoshita T, Norisada M, Masumori M, Kojima K (2006). Photosynthesis and photoassimilate transport during root hypoxia in *Melaleuca cajuputi*, a flood-tolerant species, and in *Eucalyptus camaldulensis*, a moderately flood-tolerant species. Tree Physiology.

[ref-30] Kumar P, Pal M, Joshi R, Sairam RK (2013). Yield, growth and physiological responses of mung bean [*Vigna radiata* (L.) Wilczek] genotypes to waterlogging at vegetative stage. Physiology and Molecular Biology of Plants.

[ref-31] Lichtenthaler HK, Wellburn AR (1983). Determinations of total carotenoids and chlorophylls *a* and *b* of leaf extracts in different solvents. Biochemical Society Transactions.

[ref-32] Matin NH, Jalali M (2017). The effect of waterlogging on electrochemical properties and soluble nutrients in paddy soils. Paddy and Water Environment.

[ref-33] Moumita, Mahmud JA, Biswas PK, Nahar K, Fujita M, Hasanuzzaman M (2019). Exogenous gibberellic acid mitigates drought-induced damages in spring wheat. Acta Agrobotanica.

[ref-34] Müller M, Munné-Bosch S (2021). Hormonal impact on photosynthesis and photoprotection in plants. Plant Physiology.

[ref-35] Najeeb U, Bange MP, Tan DK, Atwell BJ (2015). Consequences of waterlogging in cotton and opportunities for mitigation of yield losses. AoB Plants.

[ref-36] Patel MK, Kumar M, Li W, Luo Y, Burritt DJ, Alkan N, Tran LSP (2020). Enhancing salt tolerance of plants: from metabolic reprogramming to exogenous chemical treatments and molecular approaches. Cells.

[ref-37] Rady MM, Boriek SH, El-Mageed A, Taia A, Seif El-Yazal MA, Ali EF, Hassan AAS, Abdelkhalik A (2021). Exogenous gibberellic acid or dilute bee honey boosts drought stress tolerance in *Vicia faba* by rebalancing osmoprotectants, antioxidants, nutrients, and phytohormones. Plants.

[ref-38] Rahman MM, Mostofa MG, Rahman MA, Islam MR, Keya SS, Das AK, Miah MG, Kawser AR, Ahsan SM, Hashem A, Tabassum B (2019). Acetic acid: a cost-effective agent for mitigation of seawater-induced salt toxicity in mungbean. Scientific Reports.

[ref-39] Ren B, Zhang J, Dong S, Liu P, Zhao B (2016). Effects of waterlogging on leaf mesophyll cell ultrastructure and photosynthetic characteristics of summer maize. PLOS ONE.

[ref-40] Sarafraz-Ardakani MR, Khavari-Nejad RA, Moradi F, Najafi F (2014). Abscisic acid and cytokinin-induced osmotic and antioxidant regulation in two drought-tolerant and drought-sensitive cultivars of wheat during grain filling under water deficit in field conditions. Notulae Scientia Biologicae.

[ref-41] Somogyi M (1952). Notes on sugar determination. Journal of Biological Chemistry.

[ref-42] Steffens D, Hutsch BW, Eschholz T, Losak T, Schubert S (2005). Water logging may inhibit plant growth primarily by nutrient deficiency rather than nutrient toxicity. Plant, Soil and Environment.

[ref-43] Tewari S, Mishra A, Ahmad P, Ahanger MA, Singh VP, Tripathi DK, Alam P (2018). Flooding stress in plants and approaches to overcome. Plant Metabolites and Regulation Under Environmental Stress.

[ref-44] USAID Agricultural Extension Support Activity (2017). Performance of mungbean in the south-central region of Bangladesh. http://www.aesabd.org/download/AESA-Mungbean-Study-Report-Oct-2017.pdf.

[ref-45] Wang YH, Zhang G, Chen Y, Gao J, Sun YR, Sun MF, Chen JP (2019). Exogenous application of gibberellic acid and ascorbic acid improved tolerance of okra seedlings to NaCl stress. Acta Physiologiae Plantarum.

[ref-46] Xiao Y, Wu X, Sun M, Peng F (2020). Hydrogen sulfide alleviates waterlogging-induced damage in peach seedlings via enhancing antioxidative system and inhibiting ethylene synthesis. Frontiers in Plant Science.

[ref-47] Xu QT, Yang L, Zhou ZQ, Mei FZ, Qu LH, Zhou GS (2013). Process of aerenchyma formation and reactive oxygen species induced by waterlogging in wheat seminal roots. Planta.

[ref-48] Yamauchi T, Colmer TD, Pedersen O, Nakazono M (2018). Regulation of root traits for internal aeration and tolerance to soil waterlogging-flooding stress. Physiologia Plantarum.

[ref-49] Yang D, Li Y, Shi Y, Cui Z, Luo Y, Zheng M, Chen J, Li Y, Yin Y, Wang Z (2016). Exogenous cytokinins increase grain yield of winter wheat cultivars by improving stay-green characteristics under heat stress. PLOS ONE.

[ref-50] Yeung E, van Veen H, Vashisht D, Paiva ALS, Hummel M, Rankenberg T, Steffens B, Steffen-Heins A, Sauter M, de Vries M, Schuurink RC, Bazin J, Bailey-Serres J, Voesenek L, Sasidharan R (2018). A stress recovery signaling network for enhanced flooding tolerance in *Arabidopsis thaliana*. Proceedings of the National Academy of Sciences of the United States of America.

[ref-55] Yi-Shen Z, Shuai S, FitzGerald R (2018). Mung bean proteins and peptides: nutritional, functional and bioactive properties. Food & Nutrition Research.

[ref-51] Yu CW, Murphy TM, Lin CH (2003). Hydrogen peroxide-induced chilling tolerance in mungbeans mediated through ABA-independent glutathione accumulation. Functional Plant Biology.

[ref-52] Zhang Y, Liu G, Dong H, Li C (2020). Waterlogging stress in cotton: damage, adaptability, alleviation strategies, and mechanisms. The Crop Journal.

[ref-53] Zhishen J, Mengcheng T, Jianming W (1999). The determination of flavonoid contents in mulberry and their scavenging effects on superoxide radicals. Food Chemistry.

[ref-54] Zhou W, Chen F, Meng Y, Chandrasekaran U, Luo X, Yang W, Shu K (2020). Plant waterlogging/flooding stress responses: From seed germination to maturation. Physiologia Plantarum.

